# An Algorithm for Cold Patch Detection in the Sea off Northeast Taiwan Using Multi-Sensor Data

**DOI:** 10.3390/s90705521

**Published:** 2009-07-13

**Authors:** Yu-Hsin Cheng, Chung-Ru Ho, Zhe-Wen Zheng, Yung-Hsiang Lee, Nan-Jung Kuo

**Affiliations:** Department of Marine Environmental Informatics, National Taiwan Ocean University / 2 Pei-Ning Road, Keelung 20224, Taiwan; E-Mails: B95810053@mail.ntou.edu.tw (Y.C.); D95840004@mail.ntou.edu.tw (Z.Z.); D93840002@mail.ntou.edu.tw (Y.L.); c0021@mail.ntou.edu.tw (N.K.)

**Keywords:** multi-sensors, integration filtering algorithm, cold dome, Kuroshio

## Abstract

Multi-sensor data from different satellites are used to identify an upwelling area in the sea off northeast Taiwan. Sea surface temperature (SST) data derived from infrared and microwave, as well as sea surface height anomaly (SSHA) data derived from satellite altimeters are used for this study. An integration filtering algorithm based on SST data is developed for detecting the cold patch induced by the upwelling. The center of the cold patch is identified by the maximum negative deviation relative to the spatial mean of a SST image within the study area and its climatological mean of each pixel. The boundary of the cold patch is found by the largest SST gradient. The along track SSHA data derived from satellite altimeters are then used to verify the detected cold patch. Applying the detecting algorithm, spatial and temporal characteristics and variations of the cold patch are revealed. The cold patch has an average area of 1.92 × 10^4^ km^2^. Its occurrence frequencies are high from June to October and reach a peak in July. The mean SST of the cold patch is 23.8 °C. In addition to the annual and the intraseasonal fluctuation with main peak centered at 60 days, the cold patch also has a variation period of about 4.7 years in the interannual timescale. This implies that the Kuroshio variations and long-term and large scale processes playing roles in modifying the cold patch occurrence frequency.

## 1. Introduction

**Figure 1.**
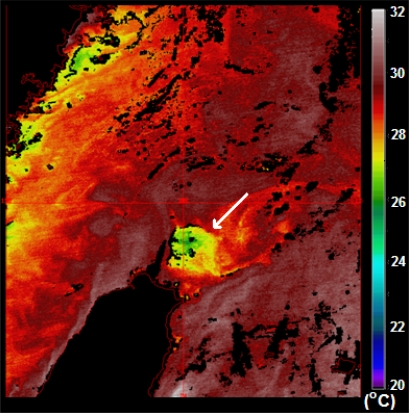
MODIS SST image taken on the 2nd August 2007. The white arrow indicates the cold patch off northeast Taiwan.

Kuroshio, the western boundary current of the North Pacific Ocean flows northward along the eastern coast of Taiwan. After leaving Taiwan, the northward flow changes direction when it runs over the continental shelf off northeast Taiwan to produce upwelling on the edge of the shelf [1–5] because of the Kuroshio on-shelf intrusion [6]. The upwelling water, seen as a cold dome, has strong temperature fluctuations and large displacements of vertical and horizontal temperature gradients. Figure 1 is a sea surface temperature (SST) image from the Moderate Resolution Imaging Spectroradiometer (MODIS) onboard the Aqua satellite showing a cold patch off northeast Taiwan. The cold patch is a sea surface feature induced by the upwelling. Because the upwelling structure may modify the sound speed and lead to uncertainty in predicting the acoustic propagation as well as being the place where exchanges of the Kuroshio Water and the East China Sea Shelf Water occur, its formation and characteristics have been drawn the attention of the scientific community.

Previous studies, including satellite observations, *in-situ* measurements, and numerical models suggested that the season of existence of the upwelling is still controversial. Lin *et al.* [8] analyzed 118 SST images and found that the cold patch on the sea surface could be identified in 96 images. The cold patch induced by the upwelling can occupy over 3,700 km^2^ area on the sea surface and reach a 6 °C difference from its coldest center to its surrounding waters. It seems that the upwelling exists all year round, but its scale, configuration, temperature distribution vary with time. Liu *et al.* [9] used chemical hydrographic data and found that the upwelling occurs year-round, but only occasionally reaches the sea surface to form a cold patch. Tang *et al.* [10] collected current observations with shipboard Acoustic Doppler Current Profilers (ADCP) between 1995 and 1997 and found the upwelling in summer, but not in winter because of the seaward and the shoreward movements of Kuroshio. Using satellite SST images, Tseng *et al.* [11] revealed the presence of a cold patch off northeast Taiwan in summer. A recent numerical study [12] showed that the upwelling exists year-round only below 150 m. In the surface layer, the upwelling occurs heavily in summer rather than in winter, because of the migration of the mean Kuroshio axis seaward in summer.

**Figure 2.**
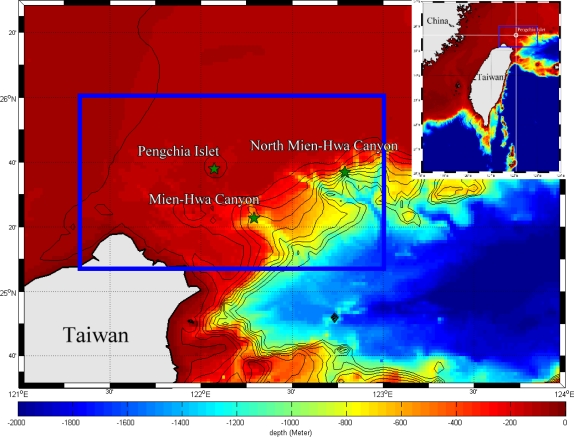
The bathymetry northeast off Taiwan. The inside of blue rectangle is the study area with bathymetry in color.

Although previous studies had stated that the formation of an upwelling is related to the movement of the Kuroshio axis, a definition of the cold patch was not clearly made. To identify and detect an upwelling event from satellite observations, the following criteria and definition are used in this study: (1) negative SST anomaly and negative SSHA are considered because of the characteristic of an upwelling region; (2) the center of the negative SST anomaly with maximum temperature drop relative to surrounding is defined as the upwelling center; and (3), the SST gradient drop from the center to the surrounding area must be large enough. The upwelling usually manifests lower SST and lower sea surface height (SSH) owing to its divergent flow pattern, therefore the SST data is used to identify the upwelling shown as a cold patch on the sea surface and the SSHA data is applied to extract the information about the upwelling beneath the sea surface [13]. The occurrence frequency, migration, and size variation of the cold patch are further investigated according to the detecting algorithm. The study area is restricted from 25.25°N to 26°N and from 121.6°E to 123°E around the Pengchia Islet as shown in Figure 2. The paper is organized as follows. The multi-sensor data are introduced in Section 2. The detecting algorithm of cold patch is described in Section 3. Analysis results and discussion are presented in Section 4. Finally, the concluding remark is made in Section 5.

## 2. Multi-Sensor Data

Multi-sensor data of SST derived from infrared and microwave radiometers from National Oceanic and Atmospheric Administration (NOAA) and Earth Observation System (EOS) series satellites are used to detect the cold patch. SSHA data derived from altimeters onboard TOPEX/Poseidon and Jason-1 are further employed to verify the detected cold patch.

### 2.1. Sea Surface Temperature

The SST data used in this study is the new generation sea surface temperature (NGSST) dataset. NGSST is a satellite-based SST product, which is generated by merging satellite SST observation from infrared radiometers of Advanced Very High Resolution Radiometer (AVHRR) onboard the NOAA series satellites and MODIS onboard Terra and Aqua satellites, as well as a microwave radiometer of Advanced Microwave Scanning Radiometer (AMSR) onboard EOS satellites. Although infrared SST has a better spatial resolution than those of microwave radiometer, the study area is often obstructed by clouds so that infrared SST images good enough for cold patch detection are seldom available. With the combination data of infrared and microwave radiometers, the spatial and temporal coverage of NGSST were substantially improved for this study. The daily NGSST dataset has been gridded at 0.05° through an optimum interpolation scheme with de-correlation scales of 200 km in latitude/longitude directions and covering five days in time. It is available at http://www.ocean.caos.tohoku.ac.jp/.

### 2.2. Sea Surface Height

The along track sea surface height anomaly (SSHA) data derived from the altimeter onboard the TOPEX/Poseidon (T/P) and Jason-1 satellites with 6-km spatial resolution and 10-day temporal interval were used to develop time series of sea surface variations along the ground track across the upwelling area (track 240). SSHA were computed with respect to a seven-year mean (1993–1999) and produced by the Archiving Validation and Interpretation of Satellite Data in Oceanography (AVISO) (available at http://www.aviso.oceanobs.com/). Finally, SSHA data obtained from T/P (from September 1992 to August 2002) and Jason-1 (from August 2002 to June 2008) are blended to constitute a longer-term and continuous time-latitude plot allowing us to verify the detecting of the cold patch.

## 3. Detecting Algorithm

A spatial-temporal integration filtering algorithm has been used to detect the cold patch from SST data. The development of this algorithm is based on two observational facts: 1) the upwelling is an area where its temperatures are lower than surrounding waters; and 2) the spatial gradient of SST is large at the boundary of the cold patch. Firstly, we define a spatial integral mean of SST as:

(1) $$\bar{T}(t)=\frac{1}{S}\;\iint_{S}\;T_{i}\;\left(x,\;y,\;t\right)\mathrm{dxdy},$$

where *T_i_* is the SST at position *x*, *y* and time *t*; and *S* is the integral area, that is, the study area. The SST deviation at each position is as:

(2) $$T_{i}^{\prime }(x,\;y,\;t)=T_{i}\;(x,\;y,\;t)-\bar{T}(t).$$

Moreover, the temporal integral mean of SST deviation is defined as:

(3) $${\bar{T}}_{i}^{\prime }(x,\;y)=\frac{1}{P}\;\int_{P}\;T_{i}^{\prime }\;(x,\;y,\;t)\mathrm{dt},$$

where *P* is the integral time, that is, the data period. The SST deviation related to the spatial and temporal mean is:

(4) $$D_{i}^{\prime }\;(x,\;y,\;t)=T_{i}^{\prime }\;(x,\;y,\;t)-{\bar{T}}_{i}^{\prime }\;(x,\;y).$$

The center of the cold patch is then defined as the lowest *T_i_* where *D_i_*′ is less than 0 °C in the study area. Notice that through the spatial-temporal integration method we filter out the center of the cold patch.

From satellite imagery we may find that the area around the center of the cold patch is more uniform, with low SST. There are sharp SST changes in the outer areas. Therefore we may define the boundary of the cold patch where the spatial gradient of SST is the largest. The spatial gradient of SST is defined as:

(5) $$\mathrm{GT}=\sqrt{{\left(\frac{\partial T}{\partial x}\right)}^{2}+{\left(\frac{\partial T}{\partial y}\right)}^{2}}.$$

## 4. Analysis Results and Discussion

### 4.1. Occurrence Frequency

**Figure 3.**
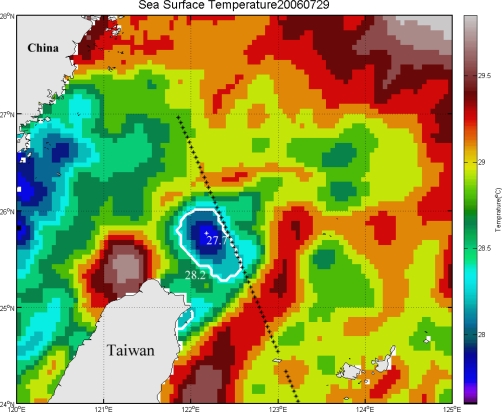
SST image of July 29, 2006. The lower SST i.e., the cold patch off northeast Taiwan is detected within the area of 27.7 °C contour (white). The black dotted line is the ground track of T/P and Jason-1.

**Figure 4.**
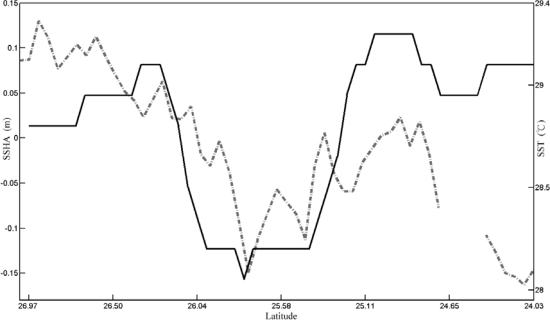
Comparison of Jason-1 sea surface height anomaly on July 28, 2006 (dashed line) and NGSST data on the July 29, 2006 (solid line) along the same transect.

The cold patch has been detected by using the aforementioned methods. Figure 3 shows an example of the cold patch which was detected by the algorithm. SST of the center is about 26.3 °C and it is 0.6 °C lower than the outside area of the cold patch. Figure 4 shows a comparison between SSHA along the altimeter track 240 (black dotted line in Figure 3) on 28th July 2006 and SST data on 29th July 2006 along the same transect. One can see that the altimeter data shows a lower SSH at 25.8°N 122.3°E which is ∼20 cm lower than the surrounding area. SST along the same transect shows a distinct temperature drop (∼1.0 °C) at almost the same region. The result not only reveals the properties of lower SST and lower SSH within the upwelling region, but also confirms the tight relationship between the negative SSHA and the cold SST in the study area. Table 1 is a statistical analysis of the cold patch detected from SST in the months from 2003 to 2008. From the yearly cold patch occurrence frequency, it seems that there is interannual variability. In 2003 and 2006, the occurrence frequency is higher than that in other years. This interannual variability implies that there are long-term and large scale processes playing roles in modifying the cold patch occurrence frequency. For the monthly cold patch occurrence frequency, the seasonal variability is obvious. The high occurrence frequencies are distributed from June to October and reach a peak in July with a maximum frequency of 53.8%. The low occurrence frequencies are distributed in winter from December to March of the next year with a minimum frequency of 1.8% in February. Other months appear to be transition period.

**Figure 5.**
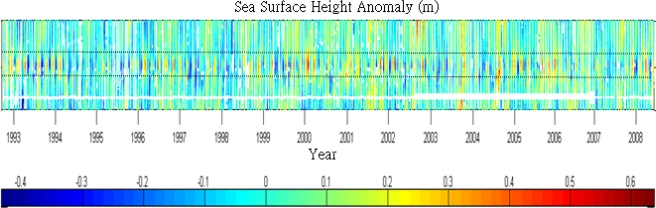
Time series of SSHA of T/P and Jason-1 along track 240 across the upwelling area. The vertical axis is the region of T/P track from northwest to southeast as shown in Figure 3. The study area is within the two dashed lines.

**Figure 6.**
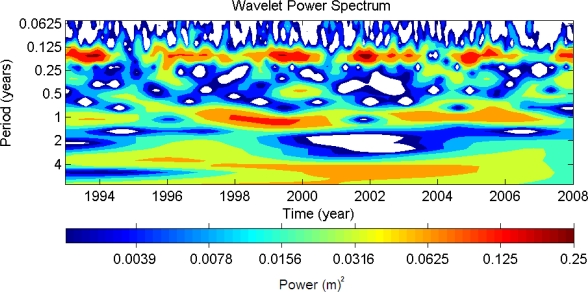
The wavelet power spectrum. The horizontal axis is the wavelet location in time and the vertical axis denotes the wavelet period in years. White patches denote power less than 0.0020 (m)^2^.

To verify the different periods of variations observed from SST, we further employ the SSHA data derived from T/P and Jason-1 altimeter data of track 240 which cross the upwelling area. Figure 5 is the time series of SSHA along the track 240 (black dotted line in Figure 3) from 1993 to 2008. The negative SSHA in cold color (blue) means the possible occurrence of cold patch characterized with lower SST and lower SSH relative to surrounding area. Compare to the ground track of altimeters and SST image shown in Figure 3, one can find that the along-track SSHA is more active within the cold patch area detected by lower SST than its outside areas. The consistent characteristics shown in both datasets confirm the detection of cold patch based on NGSST with the algorithm developed in this study. However, it is noted that the occurrence of cold patch has fluctuations in different periods. Besides the interannual and annual periods observed from SST, there are intra-seasonal periods revealed in the SSHA time series data. To get more comprehensive understanding of the variations of SSHA, wavelet transform was applied to the time series of SSHA within most active area. Figure 6 shows the power of the wavelet transform, using the Morlet-6 wavelet for the SSHA data. The horizontal axis is the wavelet location in time. The vertical axis denotes the wavelet period in years. The red patches indicate high activity areas. Clearly, three major periods including 60-day, 1-year and 4.7 years can be found in Figure 6, which reflect intra-seasonal, annual and interannual periods, respectively.

Previous study [10] suggested that the flow pattern north of Taiwan was significantly impacted by the seasonal migration of the Kuroshio. From *in-situ* measurements, they indicated that in summer Kuroshio moves away from the shelf and splits into an eastward mainstream and a northwestward branch current. At the same time, a counterclockwise circulation was found along the edge of the shelf northeast of Taiwan. On the other hand, in winter the Kuroshio moves close to and even onto the northern shelf of Taiwan and thus causes the disappearance of the counterclockwise circulation and the code patch because the intrusion of Kuroshio dominates the flow pattern in the region. Therefore, the annual period shown in the detecting result of cold patch based on SST and SSHA data are believed to be associated with the seasonal migration of Kuroshio main axis. In addition, the 60-day period is believed to be also associated with Kuroshio fluctuation because previous investigations have indicated that Kuroshio has a 60∼70-day variation [12,14]. The result of numerical modeling study [15] indicated that the high frequency variations of Kuroshio between 30 and 70 days maybe induced by the Kuroshio itself or the topographic influence on the current field. These preceding results prove that the Kuroshio variations and intrusion processes play a key role in modulating the flow pattern in this dynamic region and the occurrence frequency of cold patch. Finally, more interesting is that the 4.7-year interannual variability is firstly revealed due to the length of available observation data. The 4.7-year interannual variability implies that there are long-term and large scale processes playing roles in modifying the occurrence frequency of cold patch. However, further investigations are required to clarify the mechanism therein.

### 4.2. SST Variation

The monthly mean SST of the cold patch is listed in Table 2. Obviously, the SST displays a seasonal variation. The highest SST is in August, with a SST of 27.9 °C and the lowest SST is in March, with a SST of 19.6 °C. The mean SST of the cold patch is 23.8 °C, with a standard deviation of 2.1 °C. To remove the effect of seasonal variation, the difference of SST between the cold patch and its surrounding waters is calculated. The result is also listed in Table 2. From June to October, the SST difference is around 0.5 °C which means that SST of the cold patch is about 0.5 °C lower than its surrounding waters. In winter, the SST difference is larger than that in summer. The largest SST difference is about 0.8 °C which is shown in February. The mean SST difference is 0.6 °C with a standard deviation of 0.15 °C.

### 4.3. Centroid Movement

**Figure 7.**
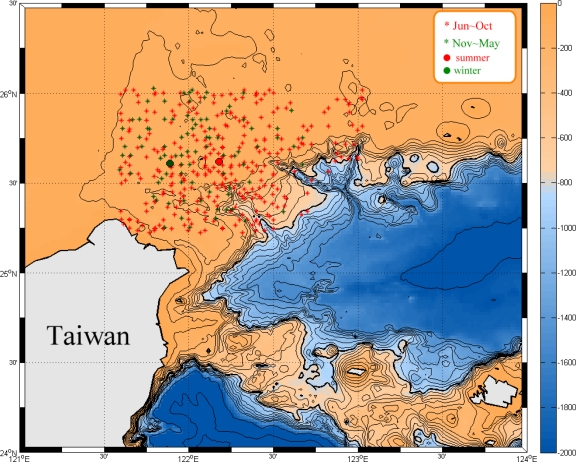
Distribution of the centroid of cold patch. Red stars denote the distribution of cold patch in summer (June to October) and green stars show the distribution of cold patch in winter and transaction periods (November to May). Red and green dots are the mean positions of distribution in summer and winter, respectively. The bathymetry is shown with contours in color.

The centroid of the cold patch is defined as the lowest SST within the upwelling area. The purpose is to understand the migration of the cold patch. Figure 7 shows the distribution of the centroid during summer (red stars), and winter and transition periods (green stars), respectively. One can see that most of them are on the continental shelf. Moreover, the centroid distribution also shows seasonal variation. Upwellings which take place during winter never distribute westward over 122.67°E. On the contrary, upwellings occur during summer time attain to over 123°E. Its mean positions are around 25.62°N 122.18°E and 25.61°N 121.89°E, respectively. Table 3 lists the monthly mean of the centriod position. It is obvious that the position doesn’t change much in latitude but it migrates from west in January to east in July and then moves back to west. This is believed to be related to the migration of Kuroshio axis which is seaward in summer and shoreward in winter.

### 4.4. Area Variation

Based on the definition of cold patch used in detecting algorithm, the areas of the cold patch are calculated. Table 3 presents the results. Clearly, the areas of the cold patch are much larger in summer than in winter. It has an average of 1.92 × 10^3^ km^2^ on the sea surface and varies from 0.76 × 10^3^ km^2^ in January to 2.45 × 10^3^ km^2^ in September. Previous study [10] suggested that the Kuroshio moves farther seaward (shoreward) in summer (winter). While the main axis of Kuroshio moves shoreward in winter, the cold patch may be compressed and even disappear. Therefore, the seasonal variations of area of the cold patch are also concluded to be associated with the seasonally zonal migration of Kuroshio. In general, the cold patch in summer is twice as large as the cold patch in winter.

## 5. Conclusions

The NGSST data merged from sensors of AVHRR, MODIS, and AMSR-E, as well as the SSHA data derived from altimeters onboard the TOPEX/Poseidon and Jason-1 satellites have been used to identify the cold patch in the sea off northeast Taiwan. A spatial-temporal integration filtering method has been developed for detecting the cold patch. The development of this method is based on two observational facts: 1) the upwelling is an area where its temperatures are lower than surrounding waters; and 2) the spatial gradient of SST is large at the boundary of the cold patch. Applying the detecting algorithm, the variations of the cold patch are further investigated. The major results are summarized as follows.

The yearly distribution of SST-observed cold patch occurrence frequencies from 2003 to 2008 reveals an interannual variability. This interannual variability is confirmed by the Wavelet analysis on SSHA data extracted form the cold patch region. The peak period of the interannual variability is around 4.7 years. This implies that there are long-term and large scale processes playing roles in modifying the cold patch occurrence frequency in the sea off northeast Taiwan. The monthly SST-observed cold patch occurrence frequencies show that high occurrence frequencies are distributed from June to October and reach a peak in July. The low occurrence frequencies are in winter, from December to April of the next year. This result shows good agreement with previous studies and is attributed to the seaward migration of the Kuroshio axis in summer and its shoreward one in winter. In addition to the seasonal and interannual variations, a 60-day variation of cold patch occurrence frequency is also found from SSHA data. Compared to previous studies, this intra-seasonal variation is also related to the Kuroshio fluctuation.

The monthly mean SST of the cold patch varies from 27.9 °C in August to 19.6 °C in March, with an average of 23.8 °C and a standard deviation of 2.1 °C. The SST difference between the cold patch and its surrounding waters also reveals seasonal variations. In general, the SST difference is around 0.5 °C in summer, but the largest difference is 0.8 °C in February. The centroid of the cold patch moves to east in summer and backs to the west in winter. The mean positions are around 25.62°N 122.18°E and 25.61°N 121.89°E, respectively. The area of the cold patch ranged from 0.76 × 10^3^ km^2^ in January to 2.45 × 10^3^ km^2^ in September, with an average of 1.92 × 10^3^ km^2^.

## Figures and Tables

**Table 1.** Number of the cold patch detected from SST in the months from 2003 to 2008.

	**Year**							

**Month**	**2003**	**2004**	**2005**	**2006**	**2007**	**2008**	**Total**	**Occur freq**

**Jan**	0	4	0	0	0	0	4	2.2%
**Feb**	0	3	0	0	0	0	3	1.8%
**Mar**	3	2	1	0	3	1	10	5.4%
**Apr**	8	1	2	3	2	4	20	11.1%
**May**	11	8	6	2	9	2	38	20.4%
**Jun**	8	16	9	11	7	4	55	30.6%
**Jul**	18	17	17	20	11	17	100	53.8%
**Aug**	20	11	7	15	14	10	77	41.4%
**Sep**	17	7	14	7	6	9	60	33.3%
**Oct**	13	6	7	19	11	3	59	31.7%
**Nov**	2	13	3	16	4	3	41	22.8%
**Dec**	3	1	0	3	0	0	7	3.8%

**Total**	103	89	66	96	67	53	474	

**Occur freq**	28.2%	24.3%	18.1%	26.3%	18.3%	14.5%		

**Table 2.** Mean and standard deviation (s.d.) of monthly SST of the cold patch and its difference to the surrounding waters (unit: °C).

	**SST of cold patch**		**SST difference**	

**Month**	**mean**	**s. d.**	**mean**	**s. d.**
Jan	19.9	1.56	0.6	0.19
Feb	20.0	1.38	0.8	0.30
Mar	19.6	0.35	0.6	0.13
Apr	22.4	0.71	0.6	0.12
May	24.3	1.06	0.6	0.16
Jun	25.9	1.24	0.6	0.13
Jul	27.6	0.94	0.5	0.16
Aug	27.9	0.88	0.5	0.13
Sep	27.1	0.90	0.5	0.14
Oct	25.2	1.09	0.5	0.16
Nov	23.4	1.23	0.5	0.10
Dec	21.9	1.15	0.6	0.23

**Table 3.** Monthly mean of the centroid position and area of the cold patch.

**Month**	**Latitude (°N)**	**Longitude (°E)**	**area (km^2^)**
Jan	25.60	121.83	756.25
Feb	25.60	121.90	1037.50
Mar	25.68	121.95	1375.00
Apr	25.70	122.00	2229.00
May	25.70	122.18	2122.70
Jun	25.56	122.21	2272.60
Jul	25.67	122.31	2365.50
Aug	25.63	122.28	2187.30
Sep	25.61	122.15	2447.00
Oct	25.61	121.96	2271.90
Nov	25.67	121.96	2091.94
Dec	25.57	121.88	1875.00

mean	25.63	122.05	1919.31
